# Knowledge, Attitude, and Practice of Primary Care Physicians Toward Common Skin Diseases in Sudan: A Cross-Sectional Study

**DOI:** 10.7759/cureus.97034

**Published:** 2025-11-17

**Authors:** Afifa M Alshaykh, Lubaba Y Ibrahim, Hajar F Mohammed, Dalia M Nasr, Samir A Bakhit, Esraa I Abdallah, Mohamed Y Ibrahim

**Affiliations:** 1 Medicine, Research and Publication Office, Dental Alumni and Students Affairs Office, Karary University, Khartoum, SDN

**Keywords:** attitude, dermatological conditions, knowledge, pcps, primary care physicians, primary healthcare, skin conditions, skin disorders, sudan

## Abstract

Background

Dermatological conditions are among the most common causes of global health consultations, ranking as the fourth leading cause of non-fatal disease burden. In Sudan, primary healthcare physicians serve as the first point of contact for patients with skin diseases. Given their vital role, this study aimed to assess primary healthcare physicians’ knowledge, management, and clinical practices regarding common skin diseases in Sudan.

Methodology

A cross-sectional study was conducted from April to September 2024 across Sudan’s states, which were clustered into five regions. We distributed a self-administered questionnaire to practicing primary care physicians through professional groups on social media platforms, and the sample was collected using a convenience sampling technique. The questionnaire consisted of four sections, namely, sociodemographic data, knowledge assessment (photo-based and multiple-choice questions), and attitude and practice sections. Data were analyzed using SPSS version 21 (IBM Corp., Armonk, NY, USA).

Results

Of the 211 targeted participants, 178 completed the questionnaire, with an 84.4% response rate, and 97 (59%) were females. The median knowledge score was 16 out of 25 (SD = ±4.7), and 99 (60.7%) showed sufficient knowledge. Most of the respondents (115, 70.6%) had three or fewer years of clinical experience, which was significantly correlated with their knowledge scores (p = 0.001). Vitiligo was the most recognized condition (153, 93.9%), while folliculitis was the least (92, 56.4%). A more positive attitude was linked to higher knowledge scores (r = 0.179, p = 0.022). Overall, 113 (64%) participants reported disease recognition as the most challenging part of their practice.

Conclusions

Although the majority of healthcare physicians demonstrated good knowledge, a notable proportion displayed gaps in disease management. This highlights the need for targeted training and integration of dermatology-focused modules in continuing medical education to aid in early disease recognition, reduce unnecessary referrals, and alleviate the burden on secondary and tertiary healthcare facilities.

## Introduction

According to the Global Burden of Disease study 2021, skin diseases ranked as the 18th leading cause of disability worldwide and the fourth significant contributor to the non-fatal disease burden [1,2]. Since 1990, the global mortality rate associated with skin diseases has increased by 71% [3]. The contribution of dermatological conditions to total morbidity imposes a significant cost on the community, placing a heavy strain on the healthcare resources and personnel [4].

In Africa, skin disorders vary from country to country and affect 21-87% of children, accounting for up to one-third of pediatricians’ and dermatologists’ outpatient consultations [5]. Furthermore, 6-24% of primary care visits were attributed to skin conditions, underscoring their frequent occurrence [6].

In Sudan, data regarding the overall incidence and prevalence of skin diseases is lacking. A study in Western Sudan, Nyala, found the prevalence of skin disease among the participants to be 92.6%, with the most common being fungal infections (32.6%), eczema (10.5%), and bacterial skin infections (10.3%) [7]. In another study from Sudan, infectious skin disease accounted for 57.1% of dermatological cases, with fungal infections being the most prevalent (62.5%), followed by viral skin infections. Allergic conditions such as dermatitis/eczema (10.5%) topped the non-infectious category [5].

Primary care physicians (PCPs) are the backbone of healthcare systems, but often lack sufficient knowledge. Studies from Yemen [8] and other low-resource settings, such as India [9], reported that many PCPs were not properly trained to diagnose and treat skin diseases, necessitating further training. Furthermore, the difference in experience between dermatologists and non-dermatologists may affect the quality of dermatologic care [10].

In 2023, the WHO stated that access to affordable and efficient primary care is essential to address the skin disease burden in low-resource settings, yet inadequate training still constitutes a great challenge [11]. In Sudan, access to specialist dermatologic care is limited, especially in rural areas; therefore, the role of PCPs in managing skin conditions becomes more critical. However, their ability to identify and effectively treat dermatologic cases remains unclear. Moreover, the high frequency of cases and low resource settings make it a priority to examine the knowledge status of PCPs, to direct future training programs, and address challenges faced by PCPs in delivering care to dermatologic cases. Therefore, this study aimed to assess the knowledge, attitude, and practices of primary healthcare doctors in Sudan regarding the diagnosis and management of common skin conditions.

## Materials and methods

Ethical approval

The Research and Publication Office, Dental Alumni and Students Affairs Office, Karary University provided approval to conduct the study (approval number: 24003). This study was registered on ClinicalTrials.gov (identifier: NCT06664489). The participants provided their consent and answered the survey anonymously.

Study design and setting

An observational, cross-sectional study was conducted in Sudan between April 2024 and September 2024. The data were collected through an online distributed survey across the 18 states of Sudan which were divided into five main regions, including Center (Khartoum, Gezira, Sinnar), North (River Nile, Northern state), East (Kassala, Gedaref, Red Sea), West (North Darfur, North Kordofan, Central Darfur, West Darfur, West Kordofan), and South (White Nile, Blue Nile, South Kordofan, South Darfur, East Darfur).

Inclusion and exclusion criteria

The study population consisted of primary care doctors above 23 years of age who worked in primary healthcare settings in Sudan during the study period. Doctors less than 23 years of age, not located in Sudan, or who did not work in primary care facilities were excluded from the study.

Sample size calculation

The prevalence of good to acceptable knowledge in a study by Alotaibi et al. (2023) [12] was used to calculate the sample size using Cochran’s sample size formula, with a 95% confidence interval and 5% margin of error. The sample size was calculated to be 211.

Sampling and data collection

Sampling Technique

This study adopted a two-stage approach to select the participants. In the first stage, the 18 states were clustered into five main areas. Then, the participants were selected using convenience sampling due to accessibility challenges.

Data Collection Tool

We used an English-language, self-administered questionnaire distributed online via Google Forms. The questionnaire was adapted from previously published literature. A small pilot test was performed, and the survey was modified accordingly. It was composed of four sections: sociodemographic, knowledge, attitude, and practice.

The knowledge section contained 10 images, and the participants were asked to recognize the disease, followed by a detailed assessment part in which five diseases were investigated concerning their diagnosis, cause, investigation, and management. Each correct answer was given a value of 1, and the total score was 25. Correct answers were chosen from multiple wrong options. The standardized images were obtained from the open-access DermNetnz.org website [13]. Regarding the attitude and practice sections, participants were asked to rate their agreement with several statements using a Likert scale (strongly agree to strongly disagree) or by selecting the preferred choice from multiple options.

Data analysis

The data were analyzed using SPSS version 21 (IBM Corp., Armonk, NY, USA). Mean, median, frequency, and standard deviation were used to describe the descriptive analysis. Chi-square, Fisher’s exact, and Spearman correlation tests were used to examine the association and correlation, while Kruskal-Wallis and Mann-Whitney U tests were used to explore the differences between variables. A p-value of 0.05 was chosen as the cut-off point of significance.

## Results

Out of 211, a total of 178 primary healthcare general practitioners were accessed, of whom 15 (8.43%) participants encountered a technical issue with the Google Forms, which caused some questions not to be displayed. This missing data was determined to be Missing Completely at Random (MCAR) and was handled using available case analysis.

Demographic characteristics

Most participants (97, 59.5%) were females and aged 23-32 years (141, 86.5%). Overall, 143 (87.7%) held a bachelor’s degree as their last qualification. Around 115 (70.6%) had one to three years of experience, while the majority (140, 85.9%) encountered only one to three dermatological cases per day (Table 1).

**Table 1:** Personal characteristics of primary healthcare doctors in Sudan.

Variable	Count (n = 163)	Percentage (n = 163)
Age in years		
23–27	66	40.5%
28–32	75	46.0%
33–37	17	10.4%
More than 37	5	3.1%
Gender		
Male	66	40.5%
Female	97	59.5%
Last qualification		
Bachelor’s degree	143	87.7%
Master’s degree	14	8.6%
Doctoral degree	6	3.7%
Years of clinical experience		
1–3 years	115	70.6%
4–6 years	25	15.3%
7–9 years	15	9.2%
10 years or more	8	4.9%
Workplace		
North of Sudan	33	20.2%
East of Sudan	49	30.1%
Center of Sudan	74	45.4%
South of Sudan	6	3.7%
West of Sudan	1	0.6%
Number of dermatologic cases per day		
1–3	140	85.9%
4–6	14	8.6%
7–9	1	0.6%
More than 10	8	4.9%

Knowledge assessment

The mean score in the 25-question knowledge assessment was 15.41, and the median was 16.0 points (SD = 4.7), indicating 61.6% correct responses. A score above or equal to 60% (15 points out of 25) was defined as the cut-off point of sufficient knowledge. Overall, 99 (60.7%) doctors had adequate knowledge. As presented in Table 2, a significant link between knowledge level and years of clinical experience (p = 0.001) was observed. However, there was no association with the rest of the demographic variables.

**Table 2:** Association between knowledge level categories and personal characteristics of primary care doctors in Sudan. The values are presented as N (%). ^a^: Fisher’s exact test was used because >20% of expected counts were <5. ^b^: Pearson’s chi-square test was used (assumptions met). P-values were considered statistically significant at p < 0.05. Significant values are denoted with *.

Variable	Insufficient, n = 64 (%)	Sufficient, n = 99 (%)	Total, n = 163	Test statistic	P-value
Age (years)					
23–27	30 (46.9%)	36 (36.4%)	66	Fisher’s exact	0.509^a^
28–32	25 (39.1%)	50 (50.5%)	75		
33–37	7 (10.9%)	10 (10.1%)	17		
More than 37	2 (3.1%)	3 (3.0%)	5		
Gender					
Male	30 (46.9%)	36 (36.4%)	66	Χ² (1) = 1.782	0.182^b^
Female	34 (53.1%)	63 (63.6%)	97		
Years of clinical experience					
1–3	55 (85.9%)	60 (60.6%)	115	Fisher’s exact	0.000^a^*
4–6	4 (6.3%)	21 (21.2%)	25		
7–9 years	1 (1.6%)	14 (14.1%)	15		
10 years or more	4 (6.3%)	4 (4.0%)	8		
Last qualification					
Bachelor degree	60 (93.8%)	83 (83.8%)	143	Fisher’s exact	0.195^a^
Master degree	3 (4.7%)	11 (11.1%)	14		
Doctoral degree	1 (1.6%)	5 (5.1%)	6		
Workplace					
North of Sudan	16 (25.0%)	17 (17.2%)	33	Fisher’s exact	0.324^a^
East of Sudan	22 (34.4%)	27 (27.3%)	49		
Center of Sudan	25 (39.1%)	49(49.5%)	74		
South of Sudan	1 (1.6%)	5 (5.1%)	6		
West of Sudan	0 (0.0%)	1 (1.0%)	1		
Number of dermatologic cases per day					
1–3	53 (82.8%)	87 (87.9%)	140	Fisher’s exact	0.142^a^
4–6	5 (7.8%)	9 (9.1%)	14		
7–9	0 (0.0%)	1 (1.0%)	1		
More than 10	6 (9.4%)	2 (2.0%)	8		

The most recognized condition in the photo quiz was vitiligo (153, 93.9%), while the least identified condition was folliculitis (92, 56.4%). Additionally, in Section two, tinea capitis was the most recognized item (127, 77.9%), while only 51 (31.3%) knew its correct management plan. Fewer than one-third of the participants (50, 30.7%) recognized the photo of miliaria, and only 44 (27%) identified its cause (Table 3).

**Table 3:** Percentage of primary healthcare doctors who correctly identified the skin conditions.

Section one			
Condition		Count (n = 163)	Percentage
Vitiligo		153	93.9%
Mycetoma		139	85.3%
Wart		131	80.4%
Chickenpox		130	79.8%
Keloid		127	77.9%
Tinea corporis		124	76.1%
Seborrheic dermatitis		116	71.2%
Cutaneous leishmaniasis		114	69.9%
Herpes zoster		93	57.1%
Folliculitis		92	56.4%
Section two			
Condition		Count (N = 178)	Percentage %
Eczema	Image	107	65.6%
	Diagnosis - clinical	70	42.9%
	Treatment - topical steroids	88	54.0%
Miliaria	Image	50	30.7%
	Cause - occlusion of exocrine sweat ducts	44	27.0%
	Treatment - resolves after a day or after changing into a cooler environment	97	59.5%
Impetigo	Image	98	60.1%
	Cause - bacteria	94	57.7%
	Treatment - both topical and oral antibiotics	72	44.2%
Tinea capitis	Image	127	77.9%
	Cause - fungi	140	85.9%
	Treatment - oral Griseofulvin for 4 weeks	51	31.3%
Scabies	Image	98	60.1%
	Diagnosis - clinical	82	50.3%
	Treatment - Permethrin cream 5%	76	46.6%

Table 4 demonstrates a significant difference in knowledge scores across years of clinical experience (p < 0.000). PCPs who had four to six years and seven to nine years of experience scored higher than the other groups. There was no difference in knowledge score concerning the doctor’s age, gender, workplace, and last qualifications.

**Table 4:** Differences in the knowledge score of primary healthcare doctors regarding common dermatological conditions in Sudan with regard to their demographic data (n = 163). P-values were considered statistically significant at <0.05. Significant values are denoted with *.

Null hypothesis	Test	Sig.	Decision
The distribution of the knowledge score is the same across categories of age	Independent-samples Kruskal-Wallis test	0.085	Retain the null hypothesis
The distribution of the knowledge score is the same across categories of gender	Independent-samples Mann-Whitney U test	0.104	Retain the null hypothesis
The distribution of the knowledge score is the same across categories of years of clinical experience	Independent-samples Kruskal-Wallis test	0.000*	Reject the null hypothesis
The distribution of the knowledge score is the same across categories of the last qualification	Independent-samples Kruskal-Wallis test	0.090	Retain the null hypothesis
The distribution of the knowledge score is the same across categories of the workplace	Independent-samples Kruskal-Wallis test	0.712	Retain the null hypothesis

Attitude

Fisher’s exact test was performed to examine the relationship between the attitude of doctors with respect to their knowledge level. Doctors’ reported confidence in their abilities to diagnose and treat dermatological conditions was associated with their knowledge sufficiency, which was statistically significant (p = 0.002). Similarly, another statistically significant relationship (p = 0.037) between knowledge sufficiency and the doctor’s reported capability of identifying and addressing the dermatological needs of their patients was noted (Table 5).

**Table 5:** Association between the attitude of primary healthcare doctors and their knowledge category regarding common dermatological conditions in Sudan (n = 163). P-values were considered statistically significant at <0.05. Significant values are denoted with *.

Attitude	Strongly disagree	Disagree	Neutral	Agree	Strongly agree	Fisher’s exact p-value
Adequate training and resources are crucial for effective dermatological care						
Sufficient (n = 99)	9	5	22	15	48	0.930
Insufficient (n = 64)	8	6	11	11	28	
General physicians are committed to continuously improving their knowledge in dermatology						
Sufficient (n = 99)	10	7	23	17	42	0.464
Insufficient (n = 64)	9	5	15	11	24	
Access to specialists is essential for providing comprehensive dermatological care						
Sufficient (n = 99)	6	4	17	25	47	0.727
Insufficient (n = 64)	5	1	12	13	33	
It is important to perform routine skin assessments as part of patient care						
Sufficient (n = 99)	6	7	26	22	38	0.298
Insufficient (n = 64)	7	2	18	9	28	
Timely consultations with dermatologists significantly impact patient outcomes						
Sufficient (n = 99)	4	3	10	25	57	0.385
Insufficient (n = 64)	7	4	10	11	32	
General physicians play a significant role in diagnosing dermatological conditions						
Sufficient (n = 99)	5	16	36	23	19	0.160
Insufficient (n = 64)	9	7	19	20	9	
I am confident in my ability to diagnose and treat dermatological conditions						
Sufficient (n = 99)	10	32	43	11	3	0.002^*^
Insufficient (n = 64)	21	16	15	8	4	
I am capable of identifying and addressing the dermatological needs of my patients						
Sufficient (n = 99)	6	20	43	16	14	0.065
Insufficient (n = 64)	13	17	19	9	6	

An influence of age (p = 0.017, significant) and years of clinical experience (p = 0.048, significant) on doctors’ self-reported capability was noted. We found no association between doctors’ demographic characteristics and their reported confidence. There was a marked association (p < 0.001) between doctors’ reported confidence and their reported capability.

Practice

The vast majority of the participants (160, 89.9%) utilized visual examination as their primary diagnostic method. Furthermore, doctors reported fungal infections as the most commonly encountered dermatological conditions (86, 48.3%), while more than half of the doctors (113, 63.5%) considered disease recognition and diagnosis as the most difficult part of their dermatological practice, and nearly half of the doctors (86, 48.3%) would ask their colleagues or seniors when faced with a challenging dermatological condition (Table 6).

**Table 6:** Distribution of responses of practices of primary healthcare doctors regarding common dermatological conditions in Sudan.

Practice	Count (n = 163)	Percentage
What methods do you primarily use to diagnose dermatological conditions?		
Visual examination	160	89.9%
Laboratory tests	7	3.9%
Dermoscopy	8	4.5%
Clinical	3	1.7%
What are the most common dermatological conditions you encounter in your practice?		
Dermatitis	48	27.0%
Parasitic infections	4	2.2%
Bacterial infections	40	22.5%
Fungal infections	86	48.3%
When you are faced with a challenging condition, what would be your course of action?		
Treat it anyway	11	6.2%
Revise resources or guidelines	30	16.9%
Ask colleagues or seniors	86	48.3%
Refer directly	51	28.7%
What is the most difficult part of your dermatological practice		
Disease recognition and diagnosis	113	63.5%
Disease treatment	40	22.5%
When to refer	25	14.0%
How often do you refer to evidence-based resources and guidelines when treating patients with dermatological conditions?		
Never	20	11.2%
Sometimes	96	53.9%
Often	41	23.0%
Always	21	11.8%
How frequently do you update your knowledge regarding dermatology?		
Never	43	24.2%
Sometimes	98	55.1%
Often	25	14.0%
Always	12	6.7%
How frequently do you refer patients to the dermatologist?		
Never	9	5.1%
Sometimes	56	31.5%
Often	67	37.6%
Always	46	25.8%

Correlation analysis

The relationship between knowledge and attitude scores was examined using Spearman correlations, where it was indicated as weak positive (r = 0.179, p = 0.022), a result close to the relationship between doctors’ confidence in diagnosing and treating common dermatological conditions and their knowledge score (r = 0.239, p = 0.002). When examining the confidence against the attitude score, there was a moderate positive correlation (r = 0.391, p < 0.001).

Regarding the frequency of patient referral to the dermatologists, doctors’ self-reported confidence was found to have a weak negative correlation with their frequency of referral decisions (r = -0.201, p = 0.007); however, knowledge score showed no significant link with referral practice (r = -0.113, p = 0.152).

## Discussion

The study aimed to assess the knowledge of PCPs in Sudan regarding the diagnosis, management, and referral practices for common dermatological disorders. In general, 99 (60.3%) doctors demonstrated sufficient knowledge, with a mean knowledge score of 15.41 out of 25, reflecting a significant gap, particularly in disease management.

We found that most PCPs have a good basic understanding of common skin disorders, and the overall level of knowledge was considered adequate. This may be explained by the fact that dermatology is an integral part of medical curricula in most Sudanese universities; however, Sudanese doctors scored relatively lower, when compared to a study in Saudi Arabia, in which 61.5% and 89.8% of the participants correctly identified skin conditions [14]. Similarly, an earlier study in the same country found that 83.6% of primary healthcare physicians had an acceptable to good level of knowledge [12]. In contrast, a study conducted by Thakkar et al. in India revealed that the majority had poor knowledge, as 88% scored 50% or less [9]. Differences in undergraduate medical education, access to postgraduate training, mentorship, and clinical exposure might explain these variations across countries. For example, 140 (85.9%) participants reported seeing only one to three cases per day.

Moreover, the study highlighted that no variables other than years of clinical experience affected the level of knowledge (p = 0.001). This correlation reinforces the assumption that experienced doctors should possess more knowledge compared to doctors with few years of clinical experience, a finding consistent with the previously cited study in Saudi Arabia, where higher knowledge correlated with advanced age and being a consultant or specialist [12]. Likewise, a study in China in 2024 found that general practitioners with more clinical experience and advanced studies demonstrated higher diagnostic abilities than less experienced general practitioners [15]. This matches the expectation that experience enhances clinical proficiency and emphasizes the importance of clinical practice and exposure rather than sole reliance on undergraduate education.

When it comes to individual dermatological cases, the image and causative agent of tinea capitis were the most identified items, 127 (77.9%) and 140 (85.9%), respectively. This may be attributed to the fact that fungal infections are the most frequently encountered dermatological conditions in studies from Sudan, accounting for 48.3% of the cases [7]; however, only 51 (31.3%) doctors correctly managed them. This suggests a considerable gap in practice, leading to inadequate management of dermatological conditions. Additionally, viral skin disorders such as herpes zoster and bacterial infections, although significant, were less frequently identified by PCPs, 93 (57.1%) and 92 (56.4%), respectively. This indicates that doctors face more difficulty in approaching less common skin conditions. This pattern aligns with epidemiological studies from Sudan, where viral and bacterial skin diseases were less frequent than fungal infections [7]. Vitiligo and mycetoma were the most commonly recognized items in the photo-only section, 153 (93.9%) and 139 (85.3%), respectively.

Our study showed that direct visual examination was the main diagnostic tool for 160 (89.9%) participants, yet many studies have highlighted its limitations. For instance, a study in Germany in 2024 observed that dermatologists were more likely to identify skin diseases in individuals with light skin than dark skin, 72.1% vs. 52.8%, through visual diagnosis (p ≤ 0.001) [16]. This indicates that skin disorders might constitute a significant challenge in darker skin tones. This is of particular importance in the context of Sudan, as most of the population is dark. Adding diagnostic tools, such as dermoscopy, to primary care can address this issue and enhance the diagnostic accuracy in dark-skinned populations.

Furthermore, 113 (63.5%) responders struggled with disease recognition and diagnosis, considering them as the most challenging aspects. It is an unexpected result because doctors scored higher in disease recognition in comparison to management. This might reflect the complexity of clinical diagnosis in real-life situations versus structured questionnaires. Whereas a similar study from West India reported management and administration aspects as the most difficult part [9]. Overall, 86 (48.3%) doctors overcame these difficulties by asking a colleague, while 51 (28.7%) preferred to refer the patients immediately to the dermatologist. This result aligns with the same Indian study in which most of the doctors managed patients by referring them to a dermatologist, using a Google search, or seeking assistance from other colleagues.

The data showed a significant but weak correlation between knowledge levels and the confidence in identification and management of skin disorders (p = 0.002), and with the perceived ability to identify and address the dermatological needs of the patients (p = 0.037), underscoring the important role of knowledge in doctors’ confidence. However, inconsistent results were observed in different studies; for instance, Bahelah et al. showed no significant association between self-perception of competency in dermatology and the ability to classify skin diseases [8]. Another study found a positive but weak correlation [14]. Taken together, these findings suggest that confidence and self-assessed competency might not be an objective reflection of knowledge. In the current study, age (p = 0.017) and years of clinical experience (p = 0.048) had a significant influence on doctors’ self-reported capability when addressing their patients’ dermatological needs, indicating that experience has a notable effect on perceived proficiency and confidence.

The findings also revealed a fair positive association between knowledge and attitude scores (r = 0.179, p-value = 0.022), supporting the idea that enhanced knowledge may promote a more positive attitude. Conversely, the negative correlation between confidence and frequency of referrals (r = -0.201, p = 0.007) suggests that more confidence results in less referral practice. However, primary healthcare is intended to reduce referrals to secondary care; therefore, unnecessary referrals might burden the healthcare system if not properly regulated. Timely dermatological consultation might provide a solution to this problem, as 125 (77%) of our participants agreed on its positive impact on their practice.

Strengths and limitations

Our study covered a wide geographical area of the country and included doctors of varying degrees of expertise. We also focused on the most encountered dermatological cases seen in regular daily practice. However, poor internet connection during the time of the study, particularly in the west, limited our access to these areas; therefore, convenience sampling was more suitable, yet this might affect the generalizability of the results. Additionally, knowledge was assessed using an image-based quiz rather than assessing real patients through physical examination; therefore, brief history scenarios were added to some questions to add more context. Finally, we used images from publicly available resources rather than being provided or reviewed by expert dermatologists, and the questionnaire was not formally validated, which might affect the precision of the measured constructs; however, a pilot test was performed to enhance clarity and ensure comprehension.

Recommendations

Considering that 102 (62%) doctors agreed on the importance of adequate training and resources, it is advised to enhance undergraduate dermatological curricula and clinical case exposure. Additionally, comprehensive, structured, and regular dermatological courses should be an integral component of continuing medical education programs to equip primary care doctors with the essential knowledge and practical skills needed to confidently tackle most of the daily encountered cases. Doctors can also benefit from periodic updates of prevalent skin disorders along with up-to-date management protocols. Furthermore, integrating telemedicine into primary care will facilitate access to specialist care, especially in rural and geographically isolated areas, to promote timely diagnosis and reduce referral practices for more common and manageable conditions.

## Conclusions

This study revealed a notable gap in dermatological knowledge, attitude, and practice among PCPs in Sudan. While most respondents demonstrated adequate knowledge, a significant proportion displayed insufficient understanding, particularly in the management of these conditions. Clinical experience was associated with higher knowledge scores, reflecting the importance of clinical exposure. These findings emphasize the need for targeted training and continued education to address these deficiencies, enhance the competence of dermatological care, and improve patient outcomes.

## Figures and Tables

**Figure 1:**
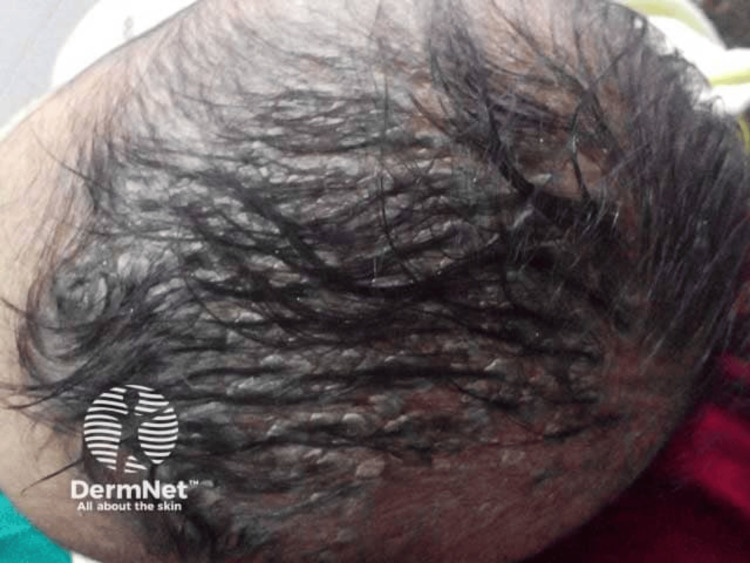
Seborrheic dermatitis on a newborn’s scalp. Erythematous, flaky plaques on the scalp of a newborn. Diagnosis: seborrheic dermatitis. Image courtesy of DermNet NZ, www.dermnetnz.org.

**Figure 2:**
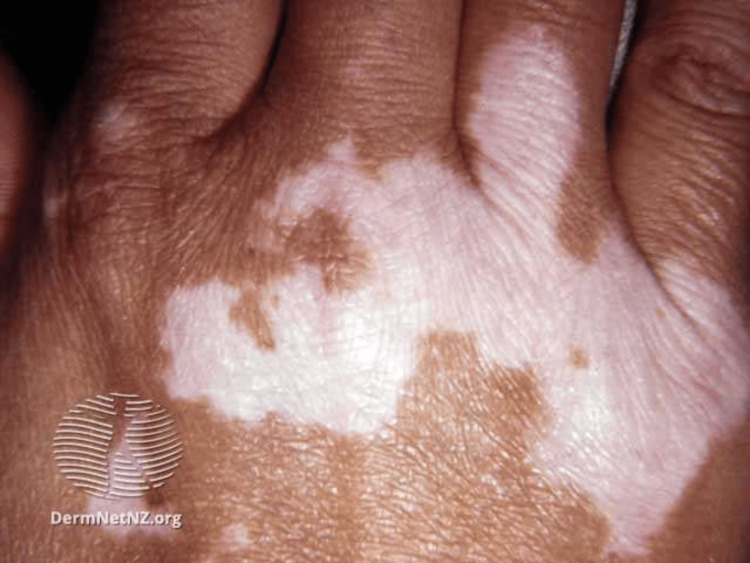
Vitiligo on the dorsal hand. Depigmented patch on the dorsal surface of the hand. Diagnosis: vitiligo. Image courtesy of DermNet NZ, www.dermnetnz.org.

**Figure 3:**
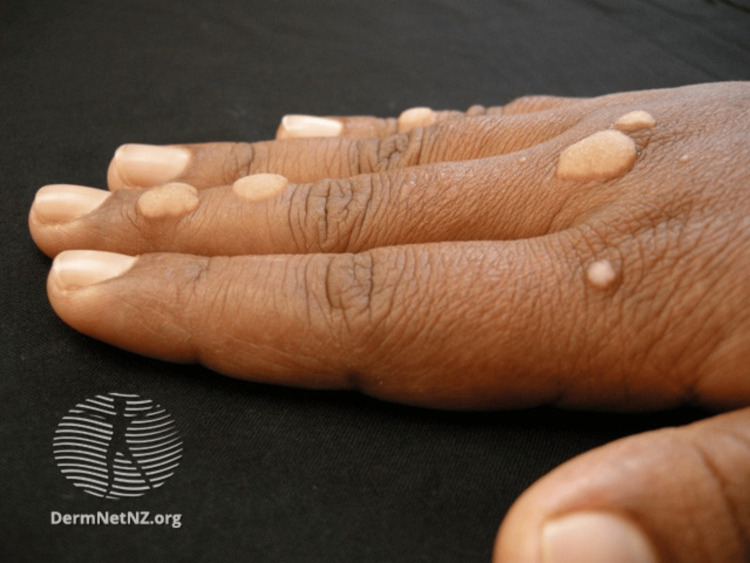
Warts on the dorsal hand Multiple papules on the dorsum of the hand. Diagnosis: warts. Image courtesy of DermNet NZ, www.dermnetnz.org.

**Figure 4:**
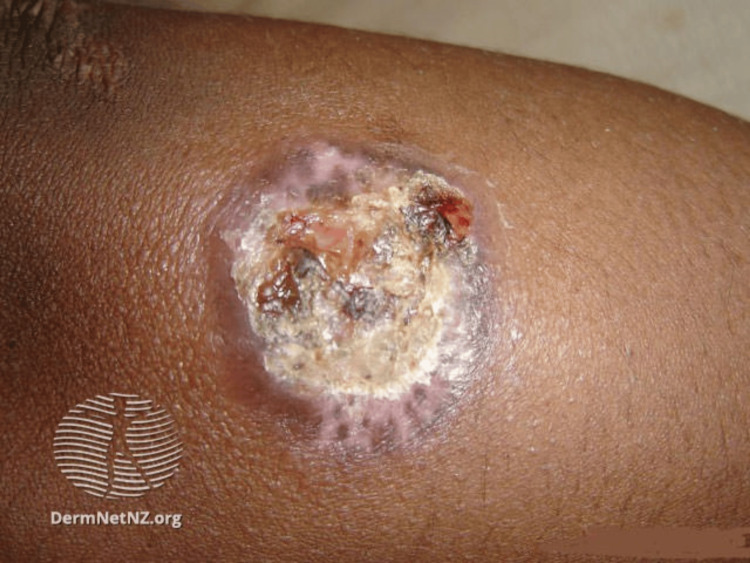
Cutaneous leishmaniasis on the arm. Ulcerated lesion on the forearm. Diagnosis: cutaneous leishmaniasis. Image courtesy of DermNet NZ, www.dermnetnz.org.

**Figure 5:**
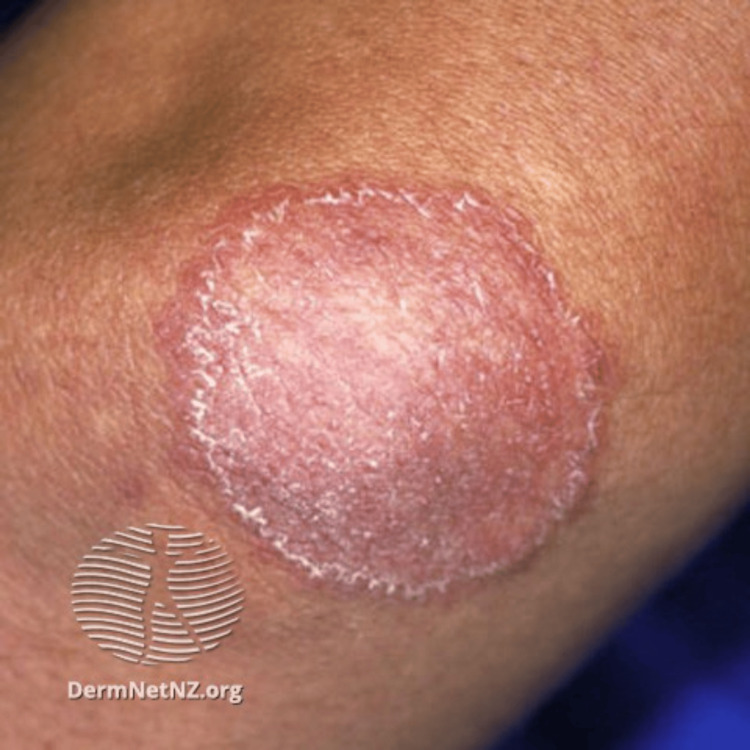
Tinea corporis. Erythematous, annular plaque with central clearing on the forearm. Diagnosis: tinea corporis. Image courtesy of DermNet NZ, www.dermnetnz.org.

**Figure 6:**
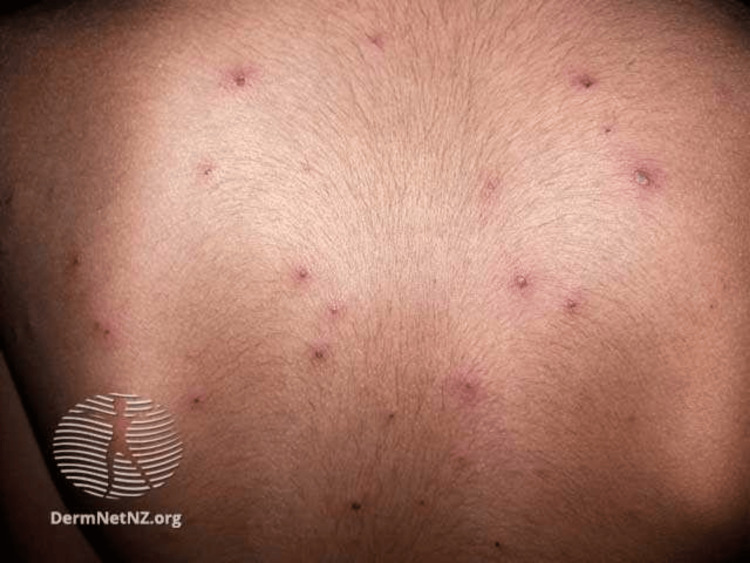
Chickenpox on the back. Multiple vesicular lesions on the back. Diagnosis: chickenpox. Image courtesy of DermNet NZ, www.dermnetnz.org.

**Figure 7:**
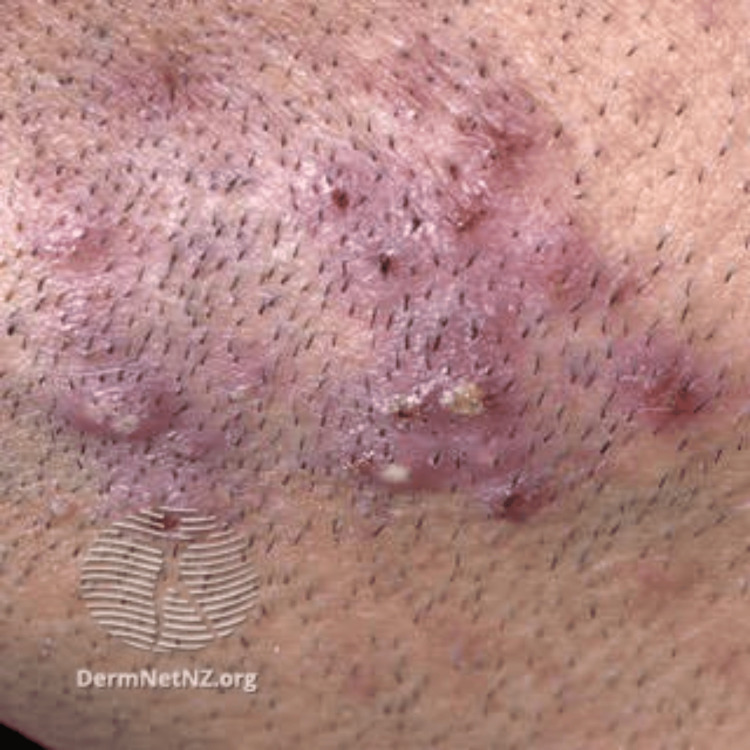
Folliculitis. Small pustules centered on hair follicles. Diagnosis: folliculitis. Image courtesy of DermNet NZ, www.dermnetnz.org.

**Figure 8:**
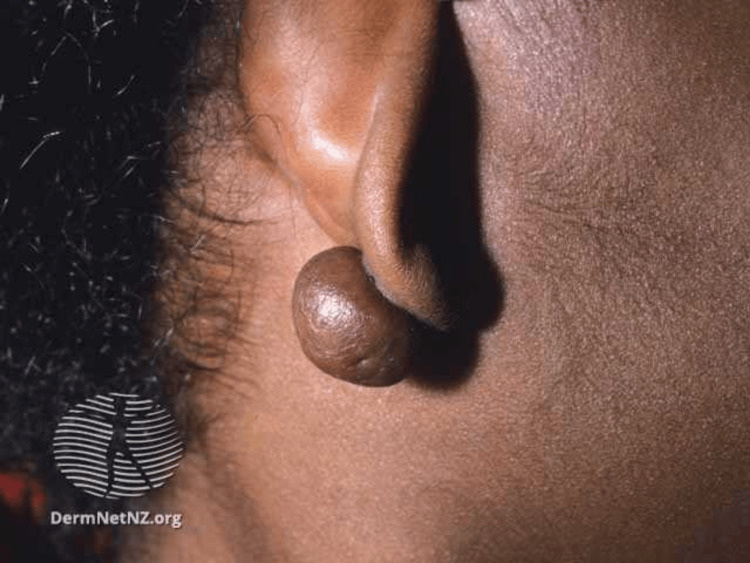
Keloid on the ear. Firm, raised scar located on the posterior ear. Diagnosis: keloid. Image courtesy of DermNet NZ, www.dermnetnz.org.

**Figure 9:**
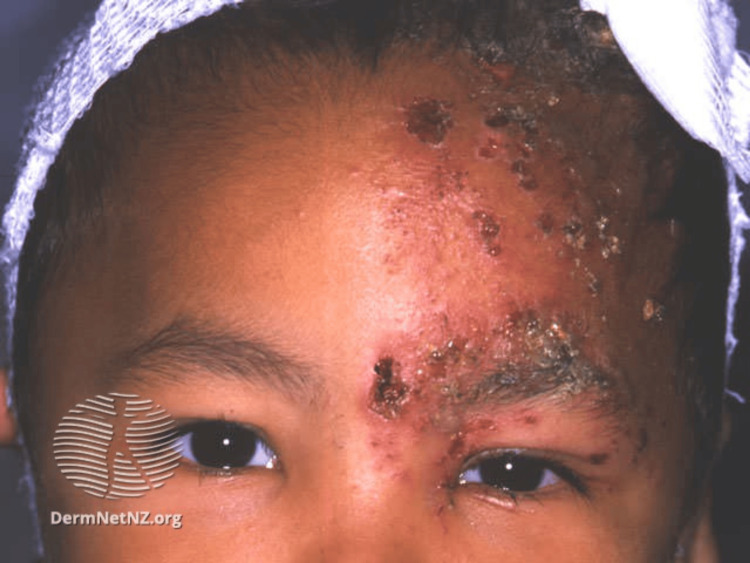
Herps zoster on the face. Vesicular lesions on the face. Diagnosis: herpes zoster. Image courtesy of DermNet NZ, www.dermnetnz.org.

**Figure 10:**
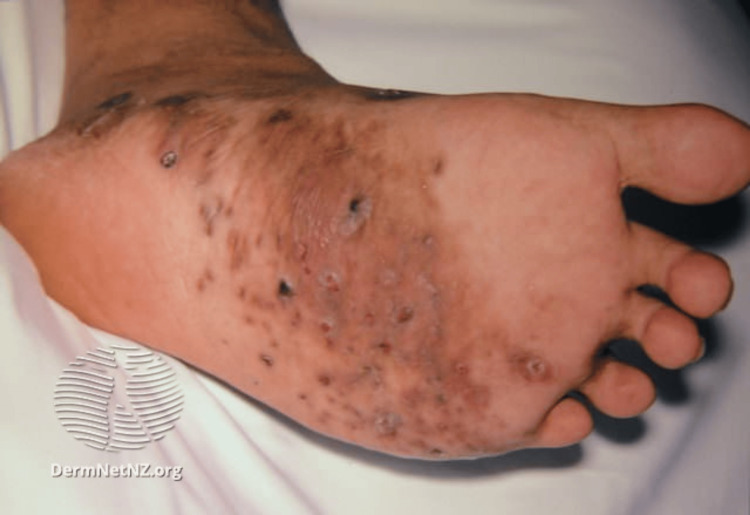
Mycetoma on the foot. Nodular swelling with draining sinuses on the foot. Diagnosis: mycetoma. Image courtesy of DermNet NZ, www.dermnetnz.org.

**Figure 11:**
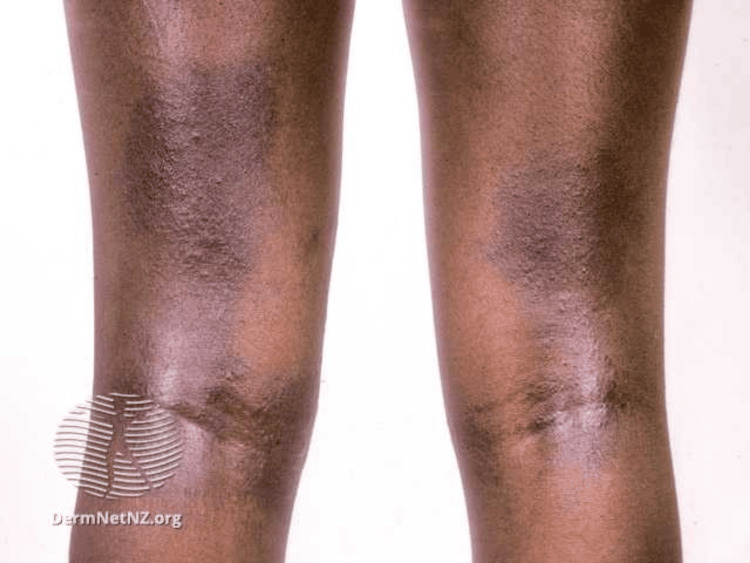
A nine-year-old girl with chronic and relapsing itching and redness in her ﬂexor regions, based on the provided image. Itchy rash and pigmentation on the back of the knee. Diagnosis: atopic dermatitis. Image courtesy of DermNet NZ, www.dermnetnz.org.

**Figure 12:**
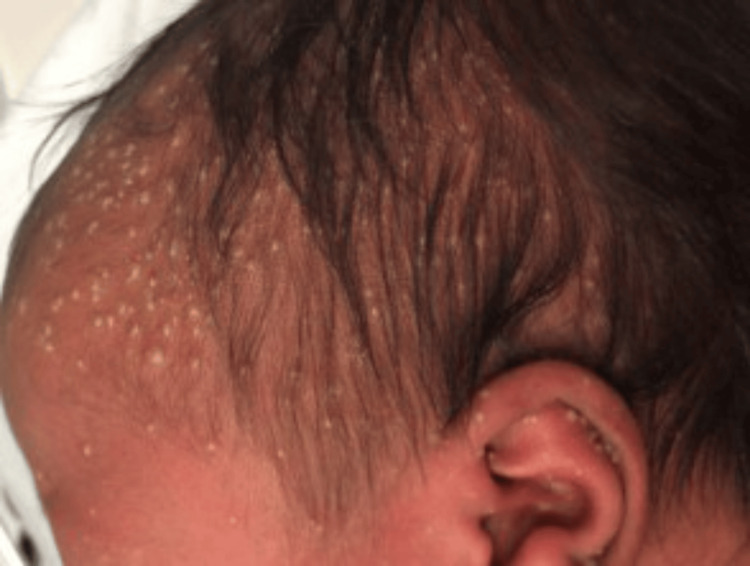
A three-week-old neonate living in a hot and humid environment brought by his mother to the clinic, with the baby in discomfort. Small papules on a newborn’s skin. Diagnosis: miliaria. Image courtesy of DermNet NZ, www.dermnetnz.org.

**Figure 13:**
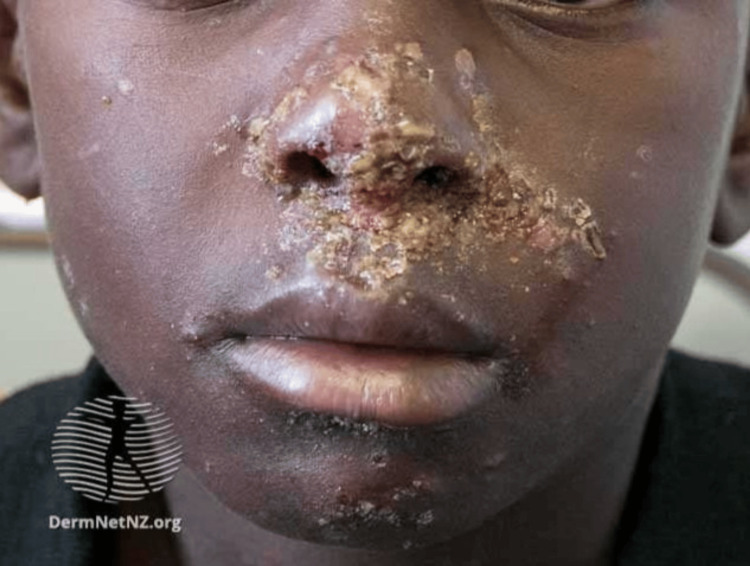
A school-aged child with lesions on the face. Honey-colored crusted lesions on the skin. Diagnosis: impetigo. Image courtesy of DermNet NZ, www.dermnetnz.org.

**Figure 14:**
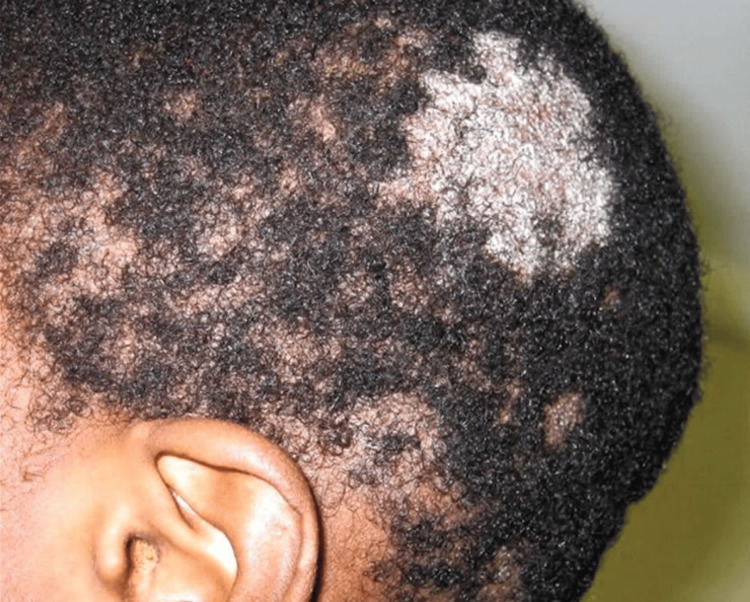
A 12-year-old boy presented to your clinic with lesion on his scalp shown in the picture. He stated that one of his schoolmate has the same lesion, based on the provided image. Scaly patches with hair loss on the scalp. Diagnosis: tinea capitis. Image courtesy of DermNet NZ, www.dermnetnz.org.

**Figure 15:**
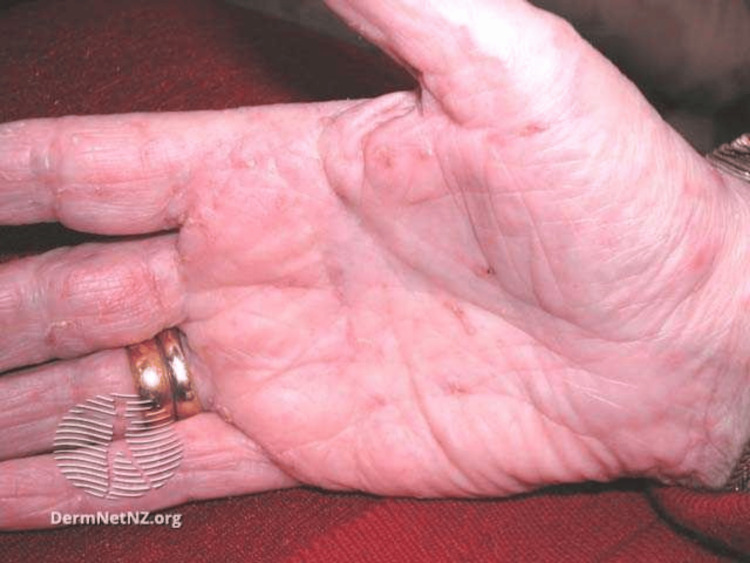
A middle-aged lady presented to the primary health center with a two-week history of skin rash involving the hands, axilla, abdomen, and genital region which is severely itchy mainly at nights. Small papules and burrows on the palm. Diagnosis: scabies. Image courtesy of DermNet NZ, www.dermnetnz.org.

## Appendices

Assessment of knowledge, attitude, and practice of primary care physicians regarding diagnosis and management of common dermatologic conditions in Sudan 2024 - Survey

1. Participation is voluntary, and all the data will remain conﬁdential and will only be used for research purposes

2. I understand and agree

Sociodemographic data

3. Age

23-27

28- 32

33- 37

More than 37

4. Gender

Male  

Female

5. Clinical experience

1-3 years

4-6 years

7-9 years

10 years or more

6. Last qualiﬁcation

Bachelor degree

Master degree

Doctoral degree

Other:

7. Workplace

River Nile state - Khartoum state - Gadaref state - Geziera state - Red Sea state - Kassala state - Northern state - Sennar state - White Nile state - Blue Nile state - South Kordofan state - North Kordofan state - South Darfur state - East Darfur state - West Darfur state - West Kordofan state - Central Darfur - North Darfur

8. Number of dermatologic cases seen per day

1-3

4-6

7-9

More than 10

Assessment of knowledge of primary care physicians regarding the diagnosis and management of common dermatologic conditions

Please identify the following skin conditions

9. Seborrheic dermatitis

Folliculitis

Eczema

I don’t know

10. Vitiligo

Tinea versicolor burn

I don’t know

Burn

11. Trauma  

Keloid

Wart 

I don’t know

 12. Tuberculosis  

Carcinoma

I don’t know

Cutaneous leishmaniasis

13.  Cutaneous leishmaniasis

Tinea corporis

Human bite

I don’t know

14. Chickenpox

Acne

Insect bites

I don’t know

15. Acne

Herpes simplex

Folliculitis

I don’t know

16. Contact dermatitis

Keloid

Hypertrophic scar

I don’t know

 17. Impetigo

 Herpes zoster

 Cellulitis

 I don’t know

18. Diabetic foot

Insect bites

Mycetoma

I don’t know

Below are images of dermatological conditions. We would like you to read the scenario, view the case, and then answer the attached questions.

19. (a) What do you believe the skin condition is consistent with?

Impetigo

Psoriasis

Atopic dermatitis

I don’t know

(b) How is this case diagnosed?

Skin prick test

Clinically

Genetic testing

I don’t know

(c) The ﬁrst line of management of this condition is

Topical calcineriumm inhibitors

Topical antihistamines

Topical steroids  

I don’t know

20. (a) Based on the provided image, what do you believe the skin condition is consistent with?

Bacterial folliculitis

Eczema

Miliaria

I don’t know

(b)  What is the underlying cause?

Occlusion of exocrine sweat ducts

Bacteria

Immune reaction

I don’t know

(c) What is the treatment of choice?

Topical antibiotics

Topical steroid

Resolve after a day or after changing into a cooler environment

I don’t know

21. (a) What do you believe the skin condition is consistent with?

Bacterial folliculitis

Impetigo

Herpes simplex

I don’t know

(b) What is the underlying cause?

Virus

Fungi

Bacteria

I don’t know

(c) What would be your initial treatment of this case?

Topical antibiotics

Topical antiviral

Cleaning the affected area with mild soap and water

Both topical and oral antibiotics

I don’t know

22. (a) What do you believe the skin condition is consistent with?

Alopecia areata

Tinea capitis

Seborrheic dermatitis

I don’t know

(b) What is the underlying cause?

Bacteria

Fungi

Immune-mediated

I don’t know

(c) What is the treatment of choice?

Topical antifungal

Topical steroids

Oral griseofulvin for 4 weeks

I don’t know

23. (a) Based on the provided image, what do you believe the skin condition is consistent with?

Papular urticaria

Eczema

Scabies

I don’t know

(b) How is this condition usually diagnosed?

Clinical diagnosis

Skin swab and culture

Skin scraping

I don’t know

(c) What is the treatment of choice?

Topical ketoconazole 2%

Permethrin cream 5%

Oral ivermectin (200 µg/kg/dose)

I don’t know

Attitude of primary healthcare doctors regarding the diagnosis and management of common dermatological conditions

Please rate your agreement with the following statements regarding attitudes toward dermatological care on a scale of 1 to 5, where 1 is strongly disagree and 5 is strongly agree

1. Adequate training and resources are crucial for effective dermatological care

2. GPs are committed to continuously improving their knowledge in dermatology

3. Access to specialists is essential for providing comprehensive dermatological care

4. I am conﬁdent in my ability to diagnose and treat dermatological conditions

5. It is important to perform routine skin assessments as part of patient care

6. Timely consultations with dermatologists signiﬁcantly impact patient outcomes 

7. I am capable of identifying and addressing the dermatological needs of my patients

8. GPs play a signiﬁcant role in diagnosing dermatological conditions 

What do you recommend to improve the level of knowledge of GPs regarding common dermatological diseases? Please select all that apply.

Publishing regular updates by the health authorities

Courses and workshops

Increasing dermatology hours at the university

Dermatology conferences

Other:

Practice of primary healthcare doctors regarding the diagnosis and management of common dermatological conditions

1. When you are faced with a challenging condition, what would be your course of action?

Treat it anyway

Revise resources or guidelines

Ask colleagues or seniors

Refer directly

Other:

2. What is the most diﬃcult part of your dermatological practice?

Disease recognition and diagnosis

Disease treatment

When to refer

Other:

3. What methods do you primarily use to diagnose dermatological conditions?

Visual examination

Laboratory tests

Dermoscopy

Other:

4. What are the most common dermatological conditions you encounter in your practice?

Dermatitis parasitic infections

Bacterial infections

Fungal infections

Other:

5. How do you typically manage dermatological conditions?

Prescribe topical medications

Prescribe oral medications

Refer to a dermatologist

Provide lifestyle advice

Other:

6. How often do you refer to evidence-based resources and guidelines when treating patients with dermatological conditions?

Never

Sometimes

Often

Always

7. How frequently do you update your knowledge regarding dermatology?

Never

Sometimes

Often

Always

8. How frequently do you refer patients to the dermatologist?

Never

Sometimes

Often

Always

## References

[1] Mengist Dessie A, Fenta Feleke S, Getaye Workie S, Getinet Abebe T, Mossu Chanie Y, Kassa Yalew A (2022). Prevalence of skin disease and its associated factors among primary schoolchildren: a cross-sectional study from a Northern Ethiopian town. Clin Cosmet Investig Dermatol.

[2] Flohr C, Hay R (2021). Putting the burden of skin diseases on the global map. Br J Dermatol.

[3] W H O (2025). World Health Organization. Skin diseases as a global public health priority. https://apps.who.int/gb/ebwha/pdf_files/WHA78/A78_R15-en.pdf.

[4] Yakupu A, Aimaier R, Yuan B (2023). The burden of skin and subcutaneous diseases: findings from the global burden of disease study 2019. Front Public Health.

[5] Ibrahim Ahmed MA, Babiker Bala SA, Ahmed Adeeel FO, Ibrahim Ahmed MA (2022). Sudanese children’s common skin diseases. J Pediatr Neonatal Care.

[6] Makaula PU, Chateau AV, Hift RJ, Dlova NC, Mosam A (2021). The impact of basic dermatology education and training on primary healthcare providers in KwaZulu-Natal, South Africa. S Afr Fam Pract (2004).

[7] Kibar Öztürk M (2019). Skin diseases in rural Nyala, Sudan (in a rural hospital, in 12 orphanages, and in two refugee camps). Int J Dermatol.

[8] Bahelah SO, Bahelah R, Bahelah M, Albatineh AN (2015). Primary care physicians’ knowledge and self-perception of competency in dermatology: an evaluation study from Yemen. Cogent Med.

[9] Thakkar SH, Chavda PD, Mehta KG (2019). Do primary care physicians require training in core clinical dermatology? A cross sectional needs assessment study from Western India. Indian J Dermatol Venereol Leprol.

[10] Fleischer AB Jr, Herbert CR, Feldman SR, O'Brien F (2000). Diagnosis of skin disease by nondermatologists. Am J Manag Care.

[11] (2025). Skin diseases as a global public health priority: no UHC without skin health for all. Skin Diseases as a Global Public Health PriorityNo UHC Without Skin.

[12] Alotaibi HM, Alruwaili ZM, Dilli AA (2023). Assessment of primary care physicians' expertise of common dermatological conditions in the Jouf region, Saudi Arabia: a mixed methods study. Healthcare (Basel).

[13] (2024). Dermatology Resource. https://dermnetnz.org/.

[14] Alsaati AA, Albejais RA, Aldawish RF (2025). Assessment of primary care physicians' ability to recognize common skin conditions in Saudi Arabia: a cross-sectional study. Cureus.

[15] Zhang L, Wang G, Chen H (2025). Diagnostic ability for common skin diseases among general practitioners working in community health service centers in Shanghai, China: a cross-sectional study. Ann Med.

[16] Krefting F, Moelleken M, Hölsken S (2024). Comparison of visual diagnostic accuracy of dermatologists practicing in Germany in patients with light skin and skin of color. Sci Rep.

